# Phytic Acid Protects from Oxidative Stress Induced by Iron-Overload and High-Fat Diets in ß2-Microglobulin Knockout Mice

**DOI:** 10.3390/molecules25225331

**Published:** 2020-11-15

**Authors:** Sixtus Aguree, Ling Guo, Manju B. Reddy

**Affiliations:** 1 Department of Food Science and Human Nutrition, Iowa State University, Ames, IA 50011, USA; saguree@iastate.edu (S.A.); ling.guo@corteva.com (L.G.); 2 Corteva Agriscience, Johnston, IA 50131, USA

**Keywords:** phytic acid, oxidative stress, NTBI, high fat diet, genetic iron overload

## Abstract

The objective of this study was to examine the protective effect of phytic acid (PA) in reducing oxidative stress in an animal model for human hereditary hemochromatosis (HH) fed high-fat diets. Sixty-four ß2 microglobulin knockout (β2m KO) mice were randomly assigned to three treatments by feeding: control (basal), atherogenic (AT), and polyunsaturated fatty acid (PUFA) diets. One-half of the mice in each treatment group were fed 2% (*wt/wt*) PA. The ß2m+/+ mice (wild type (WT)) were fed a basal diet. All seven groups were fed for 10 weeks with a 50-ppm iron-containing diet (AIN-93G). Free iron and lipids were measured in serum samples. Nonheme iron, thiobarbituric acid-reactive substances (TBARS), superoxide dismutase (SOD), and catalase concentrations were measured in the liver tissue. Nonheme iron concentration in ß2m KO mice (on the basal diet) was 20× higher (*p* < 0.0001) than in the WT mice. Compared to the WT mice, ß2m KO mice had a significantly higher concentration of free iron in the serum (*p* < 0.0001), six-fold higher hepatic TBARs (*p* < 0.0001), and 18% lower hepatic SOD level. When PA was added to the β2m KO basal diet, a reduction (26 to 50%) of iron concentration was seen in the liver and heart. The addition of PA also significantly reduced TBARs in all three dietary groups of the iron-overloaded group, but most effectively in the control group. An increase in SOD concentration was seen only in the PUFA group, but serum triacylglycerol (TG) concentration was reduced in both dietary fat groups. In conclusion, our results suggest that PA protects against oxidative stress-induced by genetic iron overload alone or when fed high fat.

## 1. Introduction

Iron is involved in many important biological functions in the body [1]. Under normal physiological conditions, the body tightly regulates iron by binding it with transferrin during transport and ferritin upon storage [2,3]. However, in iron overload conditions, such as those arising from multiple transfusions to treat anemia or hereditary hemochromatosis (HH), this delicate balance is dysregulated. HH is an autosomal recessive disorder caused by a mutation in the HFE gene [4], leading to high iron absorption, elevated plasma iron content, increased transferrin saturation, and hepatic iron overload [5,6]. In the absence of β2 microglobulin (β2m), HFE is not expressed on the membrane affecting its function in regulating iron metabolism [7] and ß2m knock out (KO) animal models show similar iron overload clinical symptoms as in HH (e.g., liver and heart) [6,8].

Since transferrin is highly saturated in overload, excess free iron exists in the form of non-protein bound or non-transferrin bound (NTBI). This free iron can be involved in the formation of free radicals by the superoxide-driven Fenton reaction [9], leading to glutathione depletion, oxidative stress, degradation of protein and nucleic acids, and lipid peroxidation [10,11]. Damaging effects of free radicals have been implicated in many diseases such as in the pathogenesis of cancer, cardiovascular diseases, and cataracts among others [12,13,14]. Antioxidants scavenge free radicals and thereby reduce the concentrations of free-radical and their related effects [15]. Phytic acid (PA), also known as myo-inositol hexaphosphate is considered an antioxidant due to its property of inhibiting hydroxyl radical formation [16,17], by chelating free iron, and making it unavailable for free radical formation [18,19]. Besides forming complexes with metal and organometallic cations in solution [20], PA has been reported to play several biological roles in living cells such as protein trafficking, cell division and differentiation, and DNA repair and protein folding [21]. Plant foods such as cereals, legumes, and oilseeds contain ubiquitous amounts of PA [22]. PA is known to provide many beneficial health effects such as reducing the risk of colonic cancer, coronary heart disease, oxidative stress, and serum cholesterol and triglyceride levels [23,24].

Phytic acid also reduces the harmful effects of oxidative stress by altering cell signaling pathways or influencing antioxidant enzymes’ expression and activities and may help prevent many types of cancers [25]. Studies have shown that dietary fat [26] and excess iron [11] alone can induce oxidative damage and increase cardiovascular disease risk. However, little is known about the combined influence of dietary fat and genetic iron overload in exacerbating oxidative stress and how PA can reduce that effect. In our study, ß2m KO mice were fed with two different high-fat diets; atherogenic (AT) and polyunsaturated fatty acid (PUFA), to investigate the oxidative stress induced by high dietary fat in an iron overload condition. We studied the effects of feeding PA on iron and lipid metabolism in iron overload ß2m KO mice fed high-fat diets. Our objective was to test the effect of PA in reducing oxidative stress and free iron concentration in iron overload in an animal model for human genetic overload.

## 2. Materials and Methods

### 2.1. Animals and Diets

**Figure 1:**
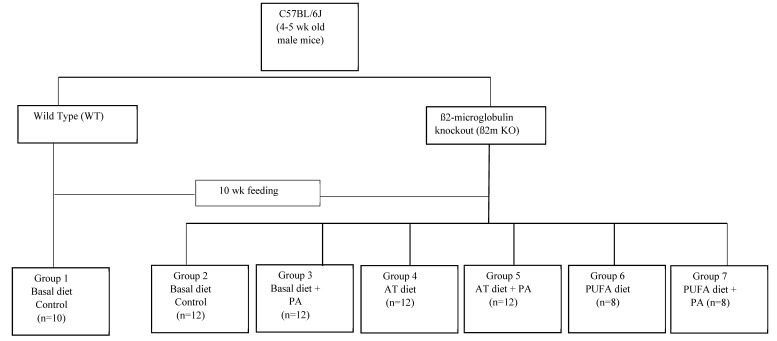
Study design.

Sixty-four four to five-week-old, male, ß2m KO mice (C57BL/6J-ß2m–/–) and ten WT mice (C57BL/6J-ß2m+/+) were obtained from Jackson Laboratory (Bar Harbor, ME, USA). The animals were housed at Iowa State University in the animal care unit in a room with a 12-h light-dark cycle. Four to five mice per cage were maintained. All procedures were approved by the Laboratory Animal Resources Committee at Iowa State University (12-7-3730-3-M). The body weight for each mouse was monitored weekly. The ß2m KO mice were randomly assigned to six different dietary groups. The WT mice fed on a basal diet served as a control. After feeding the regular mice diet for four days, all the animals were fed one of the six diets as shown in the study design (Figure 1). The animals were allowed food and water ad lib. After feeding experimental diets for ten weeks, mice were anesthetized under halothane (Sigma Chemical Company, St. Louis, MO, USA) to collect blood by heart puncture. Tissues such as the heart and liver were removed. Livers were immediately cut into small pieces, weighed, then placed into several separate tubes. All the tissue samples were kept in liquid nitrogen immediately after removal and stored at −80 °C for further analysis. Whole blood samples were centrifuged at 750× *g* for 15 min at 4 °C, and serum obtained from the whole blood was stored at −20 °C in small aliquots.

The control diet composition, AIN-93G (Harlan Tekland, Madison, WI, USA), is summarized in Table 1. The AT diet had the same composition as the basal diet, except for having 15% (*wt/wt*) fat (7.5% of soybean oil, 7.5% of cocoa butter) and 1.25% of cholesterol. The PUFA diet had the same composition as the control diet but contained 15% fat as safflower oil. To study the effect of PA in alleviating oxidative stress, 2% (*wt/wt*) of PA as sodium phytate (Sigma Chemical Company, St. Louis, MO, USA) was added to all three diets after mixing thoroughly; the WT and β2m KO fed basal diet were referred as controls.

### 2.2. Reagents and Chemicals

Trichloroacetic acid (TCA), thiobarbituric acid (TBA), phosphotungstic acid, tetraethoxypropane, nicotinamide adenine dinucleotide (NADH) and phenazine methosulfate (PMS), superoxide dismutase (SOD) standards, ethylenediaminetetraacetic acid (EDTA), nitro blue tetrazolium (NBT), phosphate-buffered saline (PBS), and 1% Triton X-100 were purchased from Sigma–Aldrich (St. Louis, MO, USA). Hydrochloric acid (HCl), acetic acid, sulfuric acid (H_2_SO_4_), magnesium chloride (MgC1_2_), sodium hydroxide (NaOH), n-butanol, and hydrogen peroxide (H_2_O_2_) were purchased from Fisher Scientific (Chicago, IL, USA). The iron standard solution for AAS was purchased from Fluka (Buchs, Switzerland).

### 2.3. Nonheme Iron Determination 

Nonheme iron concentrations in the liver and heart were measured by a modified method of Torrance and Bothwell [27]. Tissues were homogenized in water (0.1g liver/1.9 mL) and TCA (20% in 6 N HCL) was added to equal amounts of liver homogenate. The mixture was then incubated at 65 °C in the oven for 20 h, followed by centrifugation at 1400× *g* for 15 min. The supernatant was used for the nonheme iron assay with modification adapting to a microplate method.

### 2.4. Free Iron Determination 

Free iron concentration in the serum was determined by bleomycin assay [28]. All reagents were made in chelex-100 resin-treated water (Bio-Rad Laboratory, Hercules, CA, USA) in new plastic containers. A series of standards was prepared using an atomic absorption iron standard solution, diluted to give a range of standard concentrations from 0.5 to 4 µmol/L iron. The reagents were mixed together in the order as follows: 0.5 mL of 1mg/mL DNA, 0.05 mL of 1U/mL bleomycin, 0.1 mL of 50 mM MgC1_2_, 0.2 mL of Tris buffer pH 7.4, 0.05 mL of serum or iron standards, and 0.1 mL of freshly prepared ascorbic acid. The mixture was incubated in a shaking water bath at 37 °C for 1 h. The reaction was stopped by the addition of 0.1 mL of 0.1 mmol/L EDTA, followed by the addition of 0.5 mL of 1% TBA (wt/vol in 50 mmol/L NaOH) and 0.5 mL of 25% HCI. The tubes were incubated for 15 min at 100 °C to develop the color and then the absorbance was read at 532 nm. Free iron concentrations were measured against the standard curve.

### 2.5. Lipid Peroxidation

Thiobarbituric acid-reactive substances (TBARs) were measured as lipid peroxidation indicators using a modified method described by Kil et al. [29]. Liver samples were homogenized in phosphate-buffered saline (PBS, 0.9 mL/g liver) on ice with a tissue-tearor homogenizer, and the homogenates were used to determine TBARs using the following procedure. Twenty µL of liver homogenate was added to 2 mL of 40 mmol/L sulfuric acid (H_2_SO_4_); then a mixture of 0.25 mL (wt/vol) phosphotungstic acid was added and mixed. The mixture was centrifuged at 1400× *g* for 10 min. The sediment was remixed with 1 mL of H_2_SO_4_ and 0.15 mL of phosphotungstic acid and centrifuged at 1400× *g* for 10 min. The sediment was then suspended in 2 mL of distilled water and used to react with TBA. A series of standard solutions (0.125 to 1 nmol) were made by diluting with tetraethoxypropane. Then, 0.5 mL of TBA reagent (66% of TBA (wt/vol) was mixed with acetic acid in a proportion of 1:1 (vol/vol) and added to 2 mL of samples or standards and heated for 60 min at 95 °C in a water bath. When the mixture was cooled, 2.5 mL of butanol was added and shaken vigorously. The mixture was then subjected to centrifugation at 1400× *g* for 15 min, and the butanol layer was taken for the measurement of fluorescence using 553 nm emission and 515 nm excitation wavelengths (Sequoia-Turner Corporation, Mountain View, CA, USA).

### 2.6. Total Superoxide Dismutase (SOD) Activity

A nonenzymatic superoxide anion radical generation system was used to determine the SOD activity as described previously. Liver samples were homogenized on ice in 50 mmol/L phosphate buffer, pH 7.4 containing 1mM EDTA (1.9 mL/g liver). The crude homogenate was subjected to three sonication cycles of 30 pulses on ice with a 1-min interval between cycles using a Sonic Dismembrator (Fisher Scientific, Pittsburgh, PA, USA). The sample was centrifuged at 78,000× *g* for 30 min to obtain a clear extract to measure SOD. To 25 µL of test samples or SOD standards (Sigma-Aldrich Corporation, St. Louis, MO), 200 µL of freshly prepared 0.1 mmol/L EDTA, 62 µmol/L NBT, and 98 µmol/L NADH in 50mmol/L phosphate buffer, pH 7.4 were added. The reaction was started with the addition of 25 µL of freshly prepared 33 µmol/L PMS in 50 mmol/L phosphate buffer containing 0.1 mmol/L EDTA. Care was taken to add the solution quickly. An endpoint optical density was measured at 560 nm by using a microplate reader after 5 min of incubation. Percentage inhibition of O_2_-dependent NBT reduction by the sample was calculated, and the total enzyme activity was measured against the SOD standard curve. One unit is the amount of SOD required for 50% inhibition of the initial NBT reduction rate.

### 2.7. Catalase Activity

Catalase activity in the liver was measured by a method described by Abei [30] with modifications for microplate reading. Liver samples were homogenized in 50 mmol/L phosphate buffer (1 g in 0.9 mL), pH 7.0, with 1% Triton X-100. The crude homogenates were further diluted 1:250 with a phosphate buffer, pH 7.0, just before the assay. The reaction was started by adding equal amounts of H_2_O_2_, 50 mmol/L phosphate buffer pH 7.0, and samples at room temperature. The decrease in absorbance was measured at 5-s intervals for 60 s, and the first-order reaction rate constant (k1) was calculated using the software associated with the spectrophotometer. The absolute content of the enzyme in the tissue was calculated by using the following function: concentration of enzyme = reaction rate constant (k)/3.4 × 10^7^.

### 2.8. Serum Cholesterol and Triacylglycerol

Cholesterol and TG concentrations in the serum were measured using kits from Sigma (St. Louis, MO, USA) following the manufacturer’s instructions.

### 2.9. Statistical Analyses

Statistical analyses were performed using one-way ANOVA with Tukey’s multiple comparisons to determine differences among group means. The student t-test was used to test the mean differences between ß2m KO and WT mice and between ß2m KO groups with and without added PA. The differences were considered significant at *p* ≤ 0.05.

## 3. Results 

### 3.1. Weight Gain in WT and β2m KO Mice 

Percentage weight gained over 10 weeks on selected diets varied considerably across treatment groups (Table 2). Except for the PUFA diet, all animals’ groups had a mean weight gain of more than 50% at 10 weeks on a diet. There was no significant difference in percentage weight gain between the WT (86.2%) and ß2m KO (76.2%) fed the basal diet over 10 weeks. Among ß2m KO mice, those on AT (55.4%, *p* = 0.027) and PUFA (28.3%, *p* < 0.0001) diets showed a significantly smaller weight gained compared to the control group on basal diet. However, within each group of ß2m KO mice (control, AT, and PUFA), there was no difference in weight gain between mice fed with PA and without PA.

### 3.2. Iron Indices, Oxidative Stress Markers, and Serum Lipids in WT and ß2m KO Mice

Iron indices, oxidative stress markers, and the lipid profile of WT and the ß2m KO are shown in Table 3. Compared with WT, the nonheme iron concentration was 20× higher (*p* < 0.0001) in the liver and 30% lower (*p* < 0.05) in the heart of the ß2m KO mice. The free iron concentration was seven times higher (*p* < 0.0001) in ß2m KO mice compared with WT. SOD activity was 19% lower (*p* < 0.05) while catalase activity was 17% higher (*p* > 0.05) in ß2m KO mice than WT. A six-fold (*p* < 0.0001) higher lipid peroxidation, measured as TBARs, was observed in ß2m KO compared to the WT. In terms of serum lipids, only serum triacylglycerol concentrations were higher (*p* < 0001) in the ß2m KO mice than WT, but no difference in cholesterol concentration was observed.

### 3.3. Combined Effect of High-Fat Diets and Iron Overload

When a comparison was made among ß2m KO mice fed the three diets (Table 3), liver nonheme iron concentrations of mice fed an AT diet were found to be lower than in mice fed the basal diet (*p* < 0.0001) or PUFA diet (*p* < 0.001). In contrast, heart nonheme iron was lower in the mice fed the control diet (*p* = 0.019) than PUFA. There was no difference in free iron among ß2m KO mice fed high-fat diets or control, as illustrated in Table 3. The SOD activity was higher in the control than AT (*p* < 0.0001) and PUFA groups (*p* = 0.003), but catalase activity was lower in the AT group (*p* = 0.020) compared to PUFA. As indicated by TBARS, lipid peroxidation was two to three times higher with both high-fat groups than the control group (*p* < 0.001). Serum TG was about two times higher in the PUFA group compared to control (*p* = 0.0001) or AT group (*p* < 0.0001), but cholesterol was 30% higher in the AT group (*p* = 0.0042) compared to PUFA.

### 3.4. The Beneficial Effect of PA on Lowering the Consequences Associated with Iron Overload

**Figure 2:**
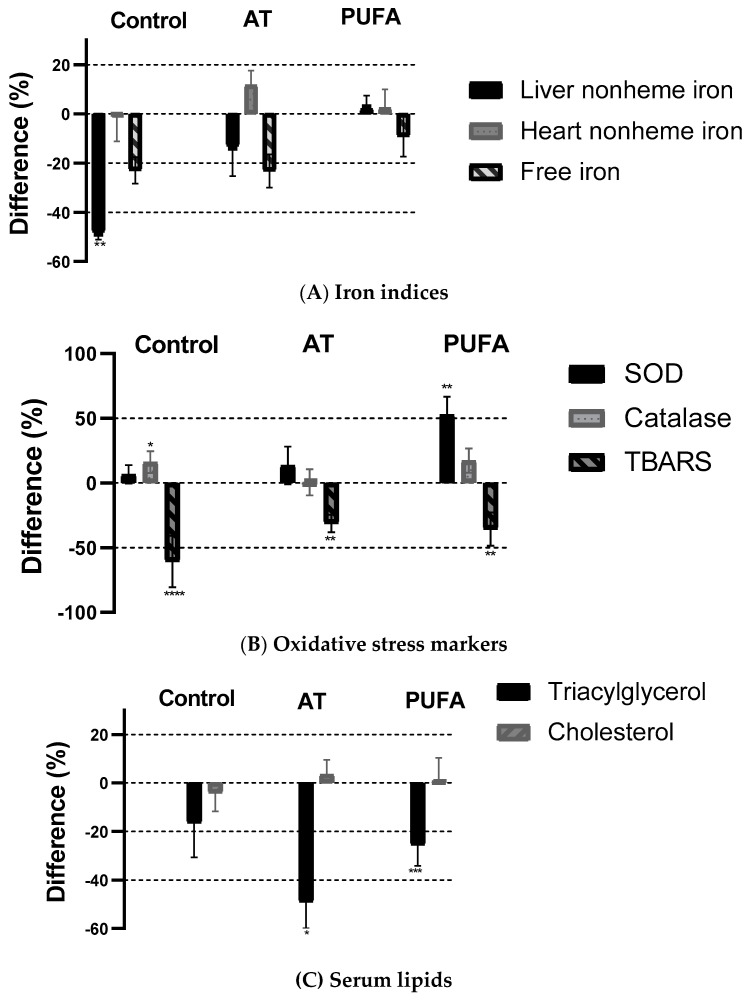
Effect of phytic acid on iron indices (**A**), oxidative stress indices (**B**), and serum lipids (**C**) fed in ß2m KO mice fed basal, AT, and PUFA diets with and without phytic acid. Each bar represents the difference (%) in value within each group (+PA/−PA * 100). The differences between with and without PA for each diet were tested using a t-test. (* *p* ≤ 0.05; ** *p* ≤ 0.01; *** *p* ≤ 0.001; **** *p* ≤ 0.001). AT, atherogenic; PUFA, polyunsaturated fatty acid; PA, phytic acid; TBARS, thiobarbituric acid-reactive substances.

Results for the three diets with added PA fed to ß2m KO mice are shown in Figure 2. A comparison was made within each group for each variable with and without added PA. The addition of 2% PA to control diets lowered the body iron burden in terms of reducing tissue iron stores. Liver nonheme iron content was 48% lower (*p* < 0.004) in the group fed the basal diet, but no significant PA effect was observed in the high-fat groups. There was no effect of PA in the heart nonheme iron concentration in any group. Free iron was 23% lower in control and AT groups but only 9% lower in the PUFA group, though none of these differences were significant (*p* > 0.05). Phytic acid was much more effective (*p* < 0.0001) in reducing TBARS (60%) in the control group but also resulted in a significant reduction in the AT and PUFA groups (*p* < 0.001). TG was significantly lower (*p* = 0.043 for AT; *p* = 0.0008 for PUFA) in the high-fat groups with added PA but not different in the control group. In all groups, we found no effect of adding PA on cholesterol concentrations.

## 4. Discussion

Phytic acid is known for its antinutritive effects, such as inhibiting mineral, especially iron absorption in humans [16,31,32] but is reported to improve blood glucose, serum lipids concentrations, attenuate iron-induced oxidative stress, and reduce lipid peroxidation [33,34,35]. We also previously showed that PA protects against 6-hydroxydopamine-induced dopaminergic neuron apoptosis in normal and iron excess conditions in a cell culture model [36], as well as decreased apoptotic cell death as measured with caspase-3 activity, and DNA fragmentation [37], suggesting its beneficial effect in Parkinson disease. In the present study, we investigated the beneficial effect of PA in terms of lowering tissue iron, especially free iron, reducing oxidative stress markers, and serum lipid concentrations, in iron overload mice when fed with AT and PUFA diets. We included both the AT and PUFA as high fat diets because one causes atherosclerosis, an underlying cause of CVD, and the other more prone to oxidative damage [38,39]. When either condition occurs in the presence of excess iron, the compounding could be harmful and increase health risks. The addition of PA was expected to lower the iron burden in both high-fat groups and protect against oxidative damage.

As expected, we found that liver iron concentration was significantly higher in β2m KO compared to WT. Our data support previous studies showing that in another iron overload model of HFe-KO (HFe−/−) mice had increased iron accumulation in the liver (five-fold higher) compared to WT [40]. Surprisingly heart iron was not higher in the β2m KO group compared to WT when fed a basal diet suggesting iron accumulation is tissue-specific, as previously reported in HFe KO [41]. These results also support previous animal studies that the liver is the most susceptible organ for iron-induced damage [42]. Similar results have also been reported in HH patients, showing the liver as the most damaged tissue due to high iron deposits [43]. In iron overload conditions such as HH, increased iron entry into plasma over time leads to saturation of serum transferrin, resulting in NTBI, a redox-active—a potentially toxic form of iron which can catalyze lipid peroxidation and cause organ damage [44,45]. In our study, higher tissue iron accumulation, free iron concentration in the serum, liver lipid peroxidation, and lower antioxidative enzymes (SOD) were observed in ß2m KO mice. The increased lipid peroxidation in the ß2m KO mice might have been related to the increased free iron concentration observed in our study. When we combined WT and ß2m KO mice data fed some basal diet, there was a strong association between free iron and lipid peroxidation (r = 0.80, *p* < 0.0001, Pearson correlation between free iron and TBARS concentrations), supporting the role of free iron in oxidative damage.

We also studied the dietary conditions that can alter the iron homeostasis in iron overload. In the present study, feeding an atherogenic diet to ß2m KO mice significantly reduced hepatic nonheme iron concentration by 3.6-fold but had no effect on the heart. While the PUFA diet did not cause significant changes in the nonheme iron concentration in the liver and heart. If liver damage occurred by feeding an atherogenic diet, it might have caused less iron storage, in which case, we expected to see higher serum iron concentration (transferrin bound) as has been reported in hemojuvelin (Hjv–/–) fed with a high-fat diet, showing a significantly higher transferrin saturation and serum iron compared to WT mice [46]. However, we were unable to test whether decreased liver iron concentrations caused an increased serum iron concentration because of the difficulty in obtaining enough serum from the mice for all the analysis we intended to perform. However, free iron did not increase in the atherogenic group, suggesting that it might not have affected serum iron.

Hepatic SOD content was also significantly lower, but TBARS were higher in the PUFA group than in the control group. This increased oxidative stress in the PUFA group may not be due to the differences in free iron concentrations because free iron concentrations were not significantly different between control and the PUFA groups. Hence, the PUFA diet enhanced oxidative stress not by altering iron stores or free iron concentrations but by providing substrate for a free radical attack as has been reported in rats fed a diet with high PUFA content [47]. In that study, rats fed on a diet with high PUFA content (salmon oil) had higher concentrations of TBARS in the liver and plasma than rat fed on a low PUFA diet (lard). The AT diet increased lipid peroxidation more than the PUFA diet. Whether this effect is by high fat alone or synergistic with iron overload is unknown, as we did not test WT mice fed the atherogenic diet. However, studies showed that dietary fat causes oxidative damage by modulating intracellular homeostasis [48], which might be through altering iron regulatory proteins (IRP) activity [49,50]. Collectively, the two high-fat diets induced oxidative stress and altered serum lipids in iron overload conditions, indicating a good model system in investigating PA beneficial effects.

To investigate dietary PA’s effect on reducing oxidative stress induced by iron overload alone or with high dietary fat, we added PA to the three diets in ß2m KO mice. We chose 2% (*wt/wt*) based on studies showing a beneficial effect of PA and at a level that does not have a detrimental effect. For example, Jariwalla [51] reported a significant reduction in bodyweight by feeding rats 9% phytate in their diets. On the other hand, PA fails to show an antioxidant effect in mice fed with 1% PA in their diet [52]. To see the beneficial effects of PA and at the same time to avoid bodyweight reduction, we chose 2% PA for each of the three diets in our study. After a 10-week feeding period, no differences in bodyweight gain were observed, indicating that dietary PA at this level did not have any negative effect on bodyweight in our study. When ß2m KO mice were fed with a PA with a basal diet, nonheme iron concentration was about 20 to 50% lower in the tissues we tested. Phytic acid consumption is well known to decrease iron absorption in humans [16] but our study did not show any alteration in free serum iron in the animal model fed three different diets. It is possible that due to the high variability in the free iron concentrations, we needed a larger sample size (number of mice per group) to detect significant differences within each treatment group. It is also possible that PA offers protection not by reducing free iron concentration, but by binding iron and makes it unavailable for free radical production [53]. A profound reduction of 60% in hepatic lipid peroxidation measured as TBARS was found with the addition of PA. A mild effect in elevated antioxidative enzymes, which were lowered in iron overload ß2m KO mice, was observed only in the control group. Our results strongly indicate that PA decreased lipid peroxidation in an iron overload condition, which would suggest its potential use in reducing consequences associated with iron overload.

Again, PA showed a reduction in the hepatic TBARs across all treatment groups, with the largest difference in the control group. Phytic acid addition improved catalase concentration in the control group, but no change was observed in the high-fat groups. Interestingly, PA addition increased hepatic SOD in the PUFA group but not in the control and AT groups. Overall, our study demonstrates the effectiveness of PA in improving hepatic SOD concentration in the PUFA group and reducing lipid peroxidation in general, as indicated by TBARS. These findings are significant because it shows that in a PUFA induced oxidative stress condition, supplementation with PA could help reduce the effect by enhancing the production of antioxidant enzymes while reducing lipid peroxidation. Since cholesterol and TG are risk factors for CVD, we also determined the beneficial effect of PA in lowering blood lipids, which is consistent with previous studies [51,54]. In this study, PA reduced serum TG by 48.9% and 25.6% in the AT and PUFA groups, respectively, but did not affect serum cholesterol content in both high-fat groups. Thus, the reduction of the oxidative damage by PA in both high-fat diets may not be due to decreasing the iron burden but may be partially due to the lowering of TG concentration. Taken together, our results indicate that PA may partially overcome the increased oxidative stress (as indicated by TBARS concentration) induced by genetic iron overload, either alone or accompanying high-fat diets. In conclusion, our studies strongly suggest that PA has excellent therapeutic potential to treat some of the consequences of iron overload, even in the presence of high-fat diets. Future studies are needed to investigate the metabolism of PA in humans and the mechanism of its protective effect in iron overload conditions.

## Figures and Tables

**Table 1:** Nutrient composition of the experimental diets (Basal, AT, and PUFA) fed to mice for 10 weeks.

	Control Diet ^1^ (g/kg)	AT Diet ^2^ (g/kg)	PUFA Diet ^2^ (g/kg)
**Casein**	200.0	200.0	200.0
**L-Cystine**	3.0	3.0	3.0
**Corn Starch**	397.5	298.3	315.8
**Maltodextrin**	132.0	132.0	132.0
**Sucrose**	100.0	100.0	100.0
**Soybean Oil**	70.0	75.0	--------
**Cocoa Butter**	--------	75.0	--------
**Cholesterol**	--------	12.5	--------
**Cholic Acid**	--------	5.0	--------
**Safflower Oil**	--------	--------	150.0
**Cellulose**	50.0	50.0	50.0
**Mineral Mix, AN-93G-MX**	35.0	35.0	35.0
**Vitamin Mix, AIN-93G-VX**	10.0	10.0	10.0
**Choline Bitartrate**	2.5	4.2	4.2
**TBHQ (Antioxidant)**	0.014	0.014	0.014

AT, atherogenic; PUFA, polyunsaturated fatty acid. ^1^ The control diet is AIN-93G, ^2^ AT and PUFA diets are modifications of the control diet.

**Table 2:** Changes in bodyweight during the 10 weeks of the experimental feeding period ^1.^

	WT	ß2m KO					
Diet	Control	Control	Control + PA	AT	AT + PA	PUFA	PUFA + PA
Initial Weight (g)	14.5 ± 0.4	17.2 ± 0.3	15.9 ± 0.5	17.5 ± 0.6	16.5 ± 0.5	22.6 ± 0.2	23.1 ± 1.2
Final Weight (g)	27.0 ± 0.3	30.3 ± 0.6	27.9 ± 0.7	27.2 ± 0.2	26.2 ± 0.4	29.0 ± 0.8	31.7 ± 0.9
% Weight Gain ^2^	86.2 ± 0.4.5	76.2 ± 3.8 ^ab^	75.5 ± 5.6 ^b^	55.4 ± 6.4 ^c^	58.9 ± 4.3 ^bc^	28.8 ± 2.8 ^d^	37.2 ± 3.0 ^de^

WT, wild type; PA, phytic acid; AT, atherogenic; PUFA, polyunsaturated fatty acid; ß2m KO, ß2-microglobulin knockout mice. ^1^ Mean ± SEM. Means differences between WT and ß2m KO control mice fed with the same basal diet were tested using a t-test. Mean differences among ß2m KO mice were analyzed using ANOVA with Tukey’s multiple comparison test. Means with different letters are significantly different (*p* < 0.05). ^2^ % wt gain = (final weight-initial weight)/initial weight) × 100.

**Table 3:** Effect of dietary fat on liver antioxidant enzyme activity and serum lipid profile in WT and β2m KO ^1.^

	WT	ß2m KO3			*p* ^2^
	Control	Control	AT	PUFA	
N	10	12	11	8	
Iron Indices					
Liver Iron (µg/g) ^4^	66 ± 5.9	1455 ± 191 ^a^	400 ± 25 ^b^	1260 ± 173 ^ac^	<0.0001
Heart Iron (µg/g) ^4^	52.6 ± 3.6	36.9 ± 5.5 ^ab^	64.1 ± 14.3 ^bc^	83.5 ± 3.6 ^c^	0.027
Free Iron (µmol/L)	3.3 ± 0.7	22.5 ± 3.2	19.1 ± 4.1	22.3 ± 2.6	<0.0001
Oxidative Stress Indices ^4^					
SOD (Unit/g) ^3^	197.8 ± 8.7	161.0 ± 12.4 ^a^	81.1 ± 6.9 ^bc^	100.9 ± 14.2 ^c^	0.030
Catalase (nmol/g)	1.03 ± 0.5	1.21 ± 0.7 ^ab^	1.05 ± 0.8 ^a^	1.3 ± 0.4 ^b^	0.056
TBARs (nmol/g)	15 ± 0.2	105 ± 10 ^a^	320 ± 28 ^b^	199 ± 11 ^c^	<0.0001
Lipid Profile ^4^					
Triacylglycerol (mg/dL)	65.6 ± 6	125.1 ±23.8 ^ab^	94.9 ± 22 ^b^	233.3 ± 10.3 ^c^	<0.0001
Cholesterol (mg/dL)	156.4 ± 7.8	155.6 ± 7.3 ^ac^	200.4 ± 13.0 ^b^	151.1 ± 3.2 ^c^	0.935

WT, wild type; AT, atherogenic; PUFA, polyunsaturated fatty acid; ß2m KO, ß2-microglobulin knockout mice; TBARS, thiobarbituric acid-reactive substances, ^1^ Mean ± SEM. Mean differences among three ß2m KO mice groups were analyzed using ANOVA with Tukey’s multiple comparison test. Values with different letters are significantly different (*p* < 0.05). ^2^ Mean difference between WT control and ß2m KO control mice fed with the same basal diet was tested using a t-test. ^3^ One unit of SOD is the amount required to inhibit the initial rate of NBT reduction by 50%. ^4^ Per gram of wet weight.
